# A functional MRI and magnetoencephalography study of the cognitive modulatory effect of transcranial direct current stimulation in early Alzheimer’s disease

**DOI:** 10.3389/fnhum.2026.1767772

**Published:** 2026-04-01

**Authors:** Himanshu Joshi, Gowthami Nair, Ashika A. Roy, Setu Havanur, Subhashini K. Rangarajan, Vanteemar S. Sreeraj, Preeti Sinha, Mariyappa Narayanan, J. Keshav Kumar, Sanjib Sinha, Jitender Saini, Sivakumar P. Thangaraju, Mathew Varghese, Paul Thompson, Ganesan Venkatasubramanian, John P. John

**Affiliations:** 1 Multimodal Brain Image Analysis Laboratory, National Institute of Mental Health and Neurosciences (NIMHANS), Bengaluru, Karnataka, India; 2 Department of Psychiatry, National Institute of Mental Health and Neurosciences (NIMHANS), Bengaluru, Karnataka, India; 3 Geriatric Clinic and Services, Department of Psychiatry, National Institute of Mental Health and Neurosciences (NIMHANS), Bengaluru, Karnataka, India; 4 Translational Psychiatry Laboratory, National Institute of Mental Health and Neurosciences (NIMHANS), Bengaluru, Karnataka, India; 5 Department of Neurology, National Institute of Mental Health and Neurosciences (NIMHANS), Bengaluru, Karnataka, India; 6 Department of Clinical Psychology, National Institute of Mental Health and Neurosciences (NIMHANS), Bengaluru, Karnataka, India; 7 Department of Neuroimaging and Interventional Radiology, National Institute of Mental Health and Neurosciences (NIMHANS), Bengaluru, Karnataka, India; 8 Imaging Genetics Center, Mark and Mary Stevens Neuroimaging and Informatics Institute, Keck School of Medicine, University of Southern California, Los Angeles, CA, United States; 9 Centre for Brain Mapping & ADBS Neuroimaging Centre, National Institute of Mental Health and Neurosciences (NIMHANS), Bengaluru, Karnataka, India

**Keywords:** Alzheimer’s disease, functional magnetic resonance imaging (fMRI), magnetoencephalography (MEG), mild cognitive impairment, transcranial direct current stimulation

## Abstract

**Objective:**

Anodal transcranial direct current stimulation (tDCS) is known to improve cognition in patients with mild cognitive impairment (MCI) and Alzheimer’s disease (mild AD).

**Methods:**

We aimed to examine the brain functional alterations accompanying improvement in cognitive performance following anodal tDCS at the left dorsolateral prefrontal cortex (DLPFC) in a sample of patients with early AD (*N* = 40; MCI, *n* = 19, and mild AD, *n* = 21) using functional magnetic resonance imaging (fMRI) and magnetoencephalography (MEG).

**Results:**

Significant (p-FDR < 0.05) reduction in seed(left middle frontal gyrus, lMFG)-to-voxel resting-state functional connectivity (rsFC) with precuneus and posterior cingulate gyrus (PCC) was noted following tDCS intervention, while task-based fMRI (tbfMRI) analysis revealed significant (p-FDR < 0.05) increases in blood oxygen level-dependent (BOLD) activations at PCC and right MFG (rMFG) during episodic memory encoding and retrieval tasks, respectively. Furthermore, a significant decrease (p-FDR < 0.05) in resting-state MEG (rsMEG) gamma power at the right occipital cortex and an increase in phase (theta) and amplitude (gamma) coupling at the left entorhinal cortex were observed post-tDCS.

**Conclusion:**

The findings of this comprehensive study using resting fMRI and MEG, as well as task-based fMRI, provide mechanistic insights regarding brain functional alterations that underlie the cognitive modulatory effects of anodal tDCS in early AD.

## 1 Introduction

Transcranial direct current stimulation (tDCS) has emerged as a safe, non-invasive brain stimulation (NIBS) procedure for enhancing cognitive functioning in mild cognitive impairment (MCI) and Alzheimer’s disease (AD) (Andrade et al., 2016; Gomes et al., 2019; Cammisuli et al., 2022; Šimko et al., 2022). Episodic memory and executive functions are key cognitive domains that are impaired in patients with MCI and mild AD (García-Herranz et al., 2016). The dorsolateral prefrontal cortex (DLPFC) plays an important role in memory and executive functions (Curtis and D’Esposito, 2003; Funahashi, 2017), and anodal stimulation at the left DLPFC improves accuracy and reaction time during episodic memory performance (Javadi and Walsh, 2012; Manenti et al., 2013) through modulation of regional electrical activity (Keeser et al., 2011). Furthermore, the cortical excitability of this region is positively associated with working memory performance (Kumar et al., 2017). A recent study reported significant improvement in general cognitive functions, immediate and delayed recall as well as new learning ability in patients with MCI, following 10 sessions of home-based anodal tDCS of the left DLFPC (Satorres et al., 2023). Thus, anodal tDCS of the left DLPFC can potentially enhance learning, memory, and executive functions in patients with MCI and mild AD. Given the feasibility of portable, home-based administration, anodal tDCS holds promise as an accessible cognition-enhancing intervention in the early stages of age-related cognitive decline, with potential relevance for community-based therapeutic and preventive strategies to delay progression to Alzheimer’s disease.

Brain functional alterations accompanying cognitive enhancement following anodal tDCS have been examined using resting and task-based fMRI. However, these studies have generated inconsistent and sometimes conflicting results, mainly due to the differences in the mode of tDCS administered (conventional vs. high definition), the site of anodal stimulation, fMRI acquisition type (resting vs. task-based), fMRI analysis methods (whole brain, seed-to-voxel, network, and voxel-wise connectivity analyses); as well as sample sizes, which very often were inadequate to derive definitive inferences from whole brain, network-based or whole-brain voxel-wise connectivity analyses (Gomes et al., 2019; Boggio et al., 2012; Das et al., 2019; Inagawa et al., 2019; Ladenbauer et al., 2017). A randomized controlled study in 43 patients with MCI involving the administration of high-definition tDCS (HD-tDCS) over the left DLPFC in the experimental group (*n* = 24) and sham tDCS in the control group (*n* = 19) showed increased intensity of the blood oxygen level dependent (BOLD) signal as measured by the fractional amplitude of low-frequency (0.01–0.1 Hz) fluctuation (fALFF) in temporo-parieto-occipital regions with a bilateral spread, and decreased signal in the right insula, precuneus, superior parietal lobe as well as left thalamus following HD-tDCS (He et al., 2021). The HD-tDCS group also showed a higher degree of synchronized oscillations measured using regional homogeneity (ReHo), in bilaterally spread fronto-temporal regions and left putamen, though no significant improvement in cognitive performance was observed following the intervention (He et al., 2021). Another study used a double-blind, cross-over, sham-controlled design in 18 patients with MCI. The authors used resting and task-based fMRI acquisitions to examine semantic word-retrieval performance along with task-related activations and voxel-level functional connectivity using eigenvector centrality mapping (ECM) during administration of anodal tDCS over the left ventral inferior frontal gyrus. Improved cognitive performance during tDCS was associated with reduction in prefrontal hyperactivity noted during the sham tDCS, increase in voxel level connectivity of prefrontal regions and reduced connectivity in more posterior brain regions (Meinzer et al., 2015). Together, these studies suggest that tDCS can alter prefrontal and distributed network dynamics, but that both the brain functional alterations and the cognitive changes associated with anodal tDCS vary across studies. A sham-controlled study with multi-session anodal tDCS at DLPFC in healthy young adults revealed reduced rsFC within DMN as well as increased rsFC between DMN and bilateral fronto-parietal network (FPN) that accompanied cognitive enhancement (Keeser et al., 2011; Peña-Gómez et al., 2012). Recent meta-analyses and systematic reviews of resting and task-based fMRI studies have reported both increased and decreased regional activations and functional connectivity in patients with subjective cognitive decline, MCI, and mild AD (Nellessen et al., 2015; Talwar et al., 2021; Wang et al., 2020) as well as following tDCS (Das et al., 2019; Chan and Han, 2020). To the best of our knowledge, no previous task-based fMRI (tbfMRI) studies in MCI or AD have reported the effect of anodal tDCS at left DLPFC on BOLD hemodynamic responses in the brain.

Electro/magnetoencephalography (EEG/MEG) gamma power and theta-gamma phase-amplitude coupling (PAC) have been established as markers of cognitive performance (Victorino et al., 2023) and have the potential to predict disease progression from MCI to AD (Goodman et al., 2018; Meinzer et al., 2022). Enhancement of working memory performance following anodal tDCS of the left DLPFC in young, healthy participants was reported to be accompanied by increased theta-gamma PAC (Ikeda et al., 2019). However, there are no reports of EEG/MEG gamma power or theta-gamma PAC alterations following anodal tDCS in MCI and AD.

Thus, evidence from extant literature for anodal tDCS-induced brain functional alterations that accompany cognitive enhancement in early AD (MCI and mild AD) is scanty and inconclusive. The reasons for the inconsistent results may include limited sample sizes in many studies, variability of tDCS (single vs. multiple sessions), and the use of different neuropsychological assessment protocols. Moreover, there is a lack of studies that have comprehensively examined such brain functional alterations, combining detailed neurocognitive assessments with resting and task-based fMRI and MEG. We aimed, therefore, to undertake such a study in a sample of patients with early AD. We hypothesized that the modulatory effect of anodal tDCS on cognitive performance in patients with early AD would be associated with alterations of rsFC and tbfMRI activation patterns, as well as enhancement of theta-gamma PAC of resting-state MEG.

## 2 Materials and methods

This study was conducted at the National Institute of Mental Health and Neurosciences (NIMHANS), Bengaluru, India, with the approval of the NIMHANS Institutional Ethics Committee. This study was registered at ClinicalTrials.gov (No. NCT06864910).

### 2.1 Study sample

All consenting participants of both sexes (age range: 55–84 years) meeting the inclusion and exclusion criteria (see below), who consecutively attended the outpatient services of the Geriatric Clinic and Services (GCS), NIMHANS from August 2019 to December 2022 with memory complaints were recruited into the study with their signed informed consent. Only right-handed individuals were recruited as assessed using the Edinburgh Handedness Inventory (EHI) (Oldfield, 1971). Participants were screened to ensure that only those with optimal hearing and visual acuity were recruited. Clinical diagnoses of MCI and mild AD made by clinicians at the GCS at NIMHANS were confirmed using the National Institute on Aging – Alzheimer’s Association (NIA-AA) diagnostic criteria (Albert et al., 2011; McKhann et al., 2011) and Clinical Dementia Rating [CDR] (Morris, 1993) (CDR scores of 0.5 for MCI and 1 for mild AD). Participants with CDR scores above 1 were excluded from this study. Other exclusion criteria included concurrent medical conditions such as hypothyroidism, hypercalcemia, vitamin B12 deficiency, uncontrolled hypertension and diabetes, neurosyphilis, normal pressure hydrocephalus, subdural haematoma, etc., requiring initiation of medical/surgical interventions and/or titration of medications; pre-existing major psychiatric and neurological illnesses, such as schizophrenia spectrum disorders, bipolar affective disorder, major depressive disorder, obsessive-compulsive disorder, substance dependence, intellectual disability disorder, Parkinson’s disease and related disorders, stroke, epilepsy, and other chronic neurological/neurodegenerative disorders, and significant head injury; and current usage of antipsychotics, antidepressants, and benzodiazepines. Forty patients (19 MCI and 21 mild AD) consented to participate in the study and completed the tDCS intervention protocol, which comprised daily 20-min tDCS sessions for 10 days. All participants underwent rsfMRI and tbfMRI, rsMEG, as well as detailed neuropsychological assessments at baseline and following tDCS intervention.

### 2.2 Clinical and cognitive assessments

The Hindi Mental Status Examination (HMSE) (Ganguli et al., 1995), a cognitive screening tool validated for the Indian population, and the Everyday Abilities Scale for India (EASI) (Fillenbaum et al., 1999), a scale validated for the Indian population for assessing activities of daily living, were administered to all participants. Detailed cognitive assessment was conducted using the NIMHANS Neuropsychological Battery for Elderly (NNB-E) (Tripathi et al., 2013). This battery includes tests of episodic memory, executive functions, attention, visuospatial function, and parietal focal signs and requires 1.5 h to administer and score. The subtests of this battery measure verbal learning and memory using a 10-word list, logical memory using a short story containing 15 facts, visuospatial learning and memory using design construction and recall with the help of small sticks, verbal and visuospatial attention using digit span and spatial span forward, working memory using digit span and spatial span backward, fluency using category, sustained attention using a picture cancellation task, planning using Tower of Hanoi. Assessments were carried out at baseline (pre-tDCS) and after completion of the last tDCS session (post-tDCS). These pre- and post-tDCS assessments were carried out by the authors HJ and SH, ensuring that the same researcher did not perform both pre- and post-tDCS assessments for a given participant, and the one carrying out the post-tDCS assessment was blind to the pre-assessment scores. Two different forms were used for the pre- and post-tDCS assessments of each participant to avoid practice effects and adequate rest between tests was provided as required (Jones, 2015; Smirni et al., 2018).

### 2.3 Transcranial direct current stimulation

Transcranial direct current stimulation was administered following standard procedures (Brunelin et al., 2012) using a neuroConn DC-Stimulator Plus device (neuroCare Group GmbH, Munich, Germany) with the anode placed at F3 (left DLPFC) and the cathode over the right supraorbital region (Fp2) using 5 × 7 cm electrodes. The anodal tDCS intervention involved daily sessions (between 10 and 11 a.m.) for 10 consecutive days, wherein a direct current (DC) of 2 mA was administered for 20 min (with additional ramp-up and ramp-down phase of 20 s each at the beginning and end of the session respectively), adhering to stringent safety measures (Brunoni et al., 2011). None of the participants reported significant adverse effects.

### 2.4 Magnetic resonance imaging

The MRIs were acquired on a 3 Tesla Philips Ingenia CX (Philips Healthcare, Netherlands) scanner with a 32-channel phased-array head coil. Participants were provided with foam pads and earplugs to minimize head motion and noise, respectively. A high-resolution structural MRI sequence was acquired in sagittal orientation with 3-D magnetization prepared rapid acquisition gradient multi-echo (MPRAGE) sequence as per the following protocol: 180 interleaved slices, repetition time [TR] = 8 ms, echo time [TE] = 3.7 ms, flip angle = 80, FOV = 240 mm^2^, 1 mm^3^ isometric voxels and acquisition time = 4 min and 40 s. During rs-fMRI acquisition, the participants were instructed to remain physically and mentally relaxed with their eyes open and not focus on anything particular. For measuring the BOLD signal, an echo-planar imaging sequence (EPI) was acquired using the following parameters: total number of volumes = 275, TR = 2,000 ms, TE = 20 ms, flip angle = 800, FOV = 240 mm^2^, 3 mm^3^ isometric voxels and acquisition time = 9 min and 24 s. Two short EPI sequences were also acquired in both the phase encoding directions to calculate the susceptibility distortion caused by magnetic field in-homogeneities during the EPI acquisition. Quality assurance (QA) of the Philips Ingenia CX scanner, as well as the quality control (QC) of the MRI data, were ensured using the standard protocol followed at the laboratory (Parekh et al., 2021).

A novel episodic memory paradigm (Gardiner, 1988) was used for tbfMRI acquisition (Supplementary Figures 1, 2). Adapted from the Sternberg (1966) item recognition task, this paradigm comprises two tasks: an incidental encoding task and an intentional retrieval task. In the incidental encoding task, the participants were instructed on each trial to indicate using a button press whether the presented visual images were of living or non-living objects, while in the intentional retrieval task, the instruction on each trial was to indicate using a button press, whether the presented visual images were seen before (memory images, MI) or not seen before (non-memory images, nMI). Before initiation of the incidental encoding task, the instruction slide showed the following: “Press ‘Index finger’ for living image and ‘Middle finger’ for non-living image. Kindly do not press any button for the ‘+’ sign.” Similarly, the instruction slide for the intentional retrieval task showed the following: “Press ‘Index finger’ for ‘seen image’ and ‘Middle finger’ for ‘not seen image’. Kindly do not press any button for the ‘+’ sign.” The visual images of living and non-living objects were taken from a pool of 260 images in the Snodgrass and Vanderwart (1980) picture collection, known for their unambiguous and nameable nature. The optimal number of trials for the incidental encoding task (living objects: 55; non-living objects: 55; fixation cross: 56) and the intentional retrieval task (memory images, MI: 55; non-memory images, nMI: 55; fixation cross: 56) was estimated using the fMRI design simulator, implemented using genetic algorithm to maximize the predictable variance among the three trial classes within each tasks (Wager and Nichols, 2003). In the incidental encoding task, 18 of the 55 trials of living images comprised 3 images (memory images; MIs) each repeated 6 times, while the remaining 37 trials comprised 37 unique images. Similarly, 11 of the 55 trials of non-living images comprised 2 MIs repeated 6 and 5 times, respectively, along with 44 unique non-living images (Supplementary Figure 1). The intentional retrieval task comprised 55 trials of 5 MIs, each repeated 11 times, and 55 trials of unique non-memory images (nMIs) that were not presented in the incidental encoding task (Supplementary Figure 2). In both tasks, no more than two successive trials of the same class were presented (Supplementary Flowchart 1). Immediately prior to the scanning session, the participants were trained to respond to the trials by following the instructions projected on the screen prior to each of the two tasks.

### 2.5 Magnetoencephalography

The pre- and post- rsMEG data were acquired using the Elekta Neuromag® TRIUX™ MEG Scanner. The scanner, installed in a magnetically shielded room, consisted of 306 sensors, including 102 magnetometers and 204 orthogonal planar gradiometers. Head digitization was done with a 3D digitizer (Fastrak Polhemus) to obtain the shape, orientation, and known locations of the head, which finally produced the 3D model of the head. The resting eyes closed MEG recordings were carried out in a sitting position employing the following protocol: sampling rate 2,000 Hz, empty room recording = 2 min, resting-state MEG = 15 min. EEG data were acquired concurrently to ensure that the participants were alert and relaxed during the recording. Electrocardiogram (ECG), electromyography (EMG), and electrooculogram (EOG) were acquired to remove the heart, muscle, and ocular artifacts, respectively. Quality control (QC) of the MEG data was done using MaxfilterTM (ver 2.2), a built-in software for the ELEKTA/Neuromag system (Elekta Neuroscience, 2010). Suppression of interference due to environmental and periodic artifacts was carried out using the spatiotemporal signal space separation (tSSS) method (Taulu and Simola, 2006).

### 2.6 Data pre-processing and analyses

#### 2.6.1 Neuropsychological performance

Two participants with mild AD were unable to complete the sustained attention and planning sub-tests. As these sub-tests are administered toward the end of the assessment battery, the overall duration of testing likely exceeded the participants’ capacity to maintain adequate attention. Despite the examiner’s efforts to re-engage them, sustained attention could not be reliably aroused, and the assessment was therefore discontinued for these sub-tests. The Shapiro Wilk test was performed to check the normality of the distribution of the differences between pre- and post-tDCS scores for each sub-test of NNB-E. Accordingly, paired *t*-tests and Wilcoxon signed rank tests were carried out, respectively, for normally and non-normally distributed sub-test scores to look for significant effects of tDCS intervention (Supplementary Table 1). Results that survived the Bonferroni correction for multiple comparisons (*p* < 0.001; *p*-value = *α*/number of comparisons) are reported as significant and others (*p* < 0.05) as trends. All the statistical analyses were performed in R version 4.0.2 “Taking Off Again” (2020) (R Core Team, 2001).

#### 2.6.2 Resting-state fMRI

The acquired MR images in Digital Imaging and Communications in Medicine (DICOM) format were converted to Neuroimaging Informatics Technology Initiative (NIfTI) format using dcm2niix version v1.0.20190902.^1^ After data anonymization, the header information for each subject was inspected and reoriented to standard format using FMRIB Software Library (FSL) (Jenkinson et al., 2012; Smith et al., 2004). The structural and functional MR images of each subject were visually examined for head motion, structural abnormalities, and scanner or acquisition-related artifacts. The functional images were further processed using DVARS (the spatial standard deviation of successive difference images) in FSL to check for volume-to-volume head movement (Power et al., 2012). The rsfMRI data of one participant each from the MCI and mild AD groups showed more than 5% intensity variation in two consecutive volumes indicating poor quality of functional images due to motion artifacts. After the exclusion of both pre- and post-rsfMRI scans of these two participants, the data of the remaining 38 participants (*n* = 18 in the MCI group and *n* = 20 in the mild AD group; Supplementary Table 2 for sociodemographic details of these participants) were further analyzed for changes in rsFC following tDCS intervention.

Analyses of rsfMRI data were carried out on CONN (version 18b) (Whitfield-Gabrieli and Nieto-Castanon, 2012), a MATLAB®^2^ based software, using statistical parametric mapping (SPM; https://www.fil.ion.ucl.ac.uk/spm/, version 7,487) in the background. A pre-processing pipeline for volume-based (with indirect normalization to MNI(2X2X2) space) analyses was implemented, which included susceptibility distortion correction using the field map, with phase and magnitude images generated using TOP-UP (Andersson et al., 2003) in FSL (Smith et al., 2004) (Supplementary material for distortion correction details). The indirect normalization pipeline involved realignment and unwarp, centering, outlier detection, indirect segmentation, normalization, and smoothing using a Gaussian kernel of FWHM at 6 mm for spatial convolution. The CONN’s default denoising pipeline was performed for subject-motion parameter estimation (Friston et al., 1996) and outlier scan identification or scrubbing (Power et al., 2012) using default threshold values (z-normalized global brain activation, movement, and rotation) in the artifact detection toolbox (ART). Using a temporal bandpass filter (0.01 Hz to 0.1 Hz), the data was denoised by regressing the principal components from white matter and CSF, determined by anatomical component-based noise correction (aCompCor) (Behzadi et al., 2007), the outliers, and the six motion parameters with their first-order derivatives and a linear term regressor.

**Figure 1:**
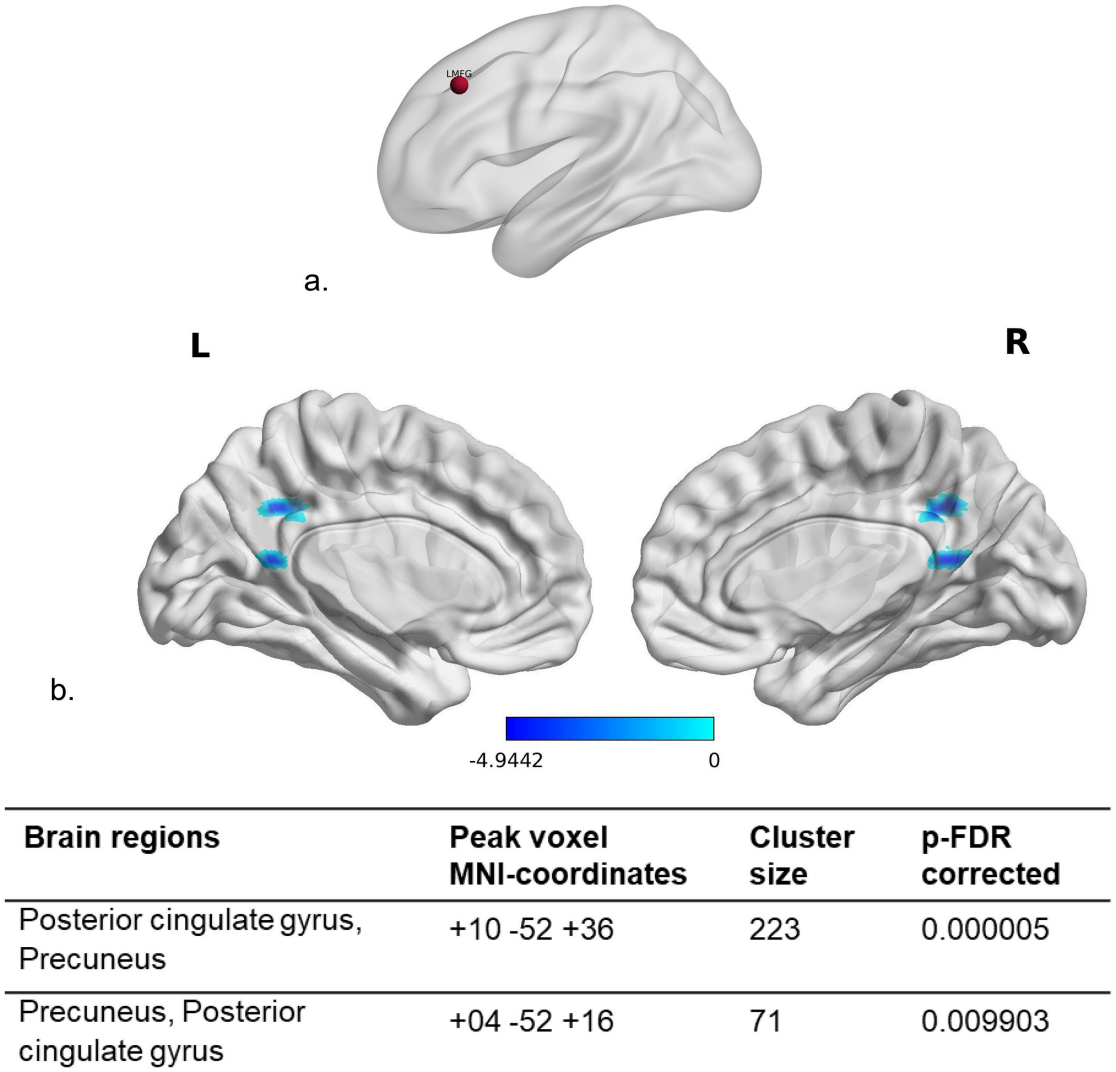
Seed-to-voxel resting-state functional connectivity (S2V-rsFC) analysis: **(a)** The dark red colored sphere indicates the location of the left middle frontal gyrus (LMFG), which was chosen as the seed for performing S2V-rsFC analysis. **(b)** The light blue-to-blue color map depicts significantly reduced S(*lMFG*)2 V-rsFC (voxel-wise *p*-uncorrected <0.001 and cluster-wise *p*-FDR < 0.05) (Chumbley et al., 2010) at bilateral posterior cingulate gyri and precuneus following tDCS intervention in the early AD [MCI (*n* = 20) + mild AD (*n* = 18)] sample. All the images are coregistered to the MNI template. Cluster coordinates are depicted as per the CONN (Whitfield-Gabrieli and Nieto-Castanon, 2012) functional connectivity toolbox and displayed using the BrainNet Viewer toolbox (Xia et al., 2013). Refer Supplementary Figure 4 for original outputs from CONN.

The seed for the seed-to-voxel resting-state functional connectivity (S2V-rsFC) analysis was set using the Harvard cortical atlas, as the left middle frontal gyrus (lMFG), corresponding to the left DLPFC, the site of the anodal tDCS. A spherical mask of size 5 mm diameter was created based on the probabilistic intensity and maximum percentage of finding this brain region (Figure 1). Seed-based connectivity (SBC) maps compute the Fisher-transformed bivariate correlation coefficients between the BOLD time series of seed and each voxel inside the brain. The analysis between pre- and post-tDCS intervention was performed using the spherical mask of the lMFG as “seed” and the whole-brain volume for “voxels.” Two-tailed parametric statistics were implemented to obtain the results using a cluster-level extent threshold (p-FDR corrected) with voxel threshold at *p* < 0.001 (*p*-uncorrected) and cluster threshold at *p* < 0.05 (*p*-corrected) (Figure 1; displayed using BrainNet Viewer toolbox (Xia et al., 2013); Supplementary material for original outputs from CONN).

#### 2.6.3 Task-based fMRI

In the incidental encoding task, “hits” were taken as the sum of responses to trials of both living and non-living images, whereas “misses” were trials that the participant missed responding. In the intentional retrieval task, the hits comprised the correct responses for trials of both previously seen and unseen images, with misses noted as trials to which the participant failed to respond. For both encoding and retrieval tasks, the participants were instructed not to press any buttons for the fixation (+) trials. False alarms were calculated as the sum of responses recorded for the fixation trials, as well as incorrect responses to the respective trials in both tasks, while correct rejections were the sum of fixation trials without responses. The probabilities of hits, misses, false alarms, and correct rejections were calculated. The inverse of standard normal cumulative distribution (ISNCD) was calculated with the probability of hits and false alarms using a NORMINV function for the calculated probability. The discriminability index (“d”) for both tasks was calculated by subtracting the ISNCD of false alarms from the ISNCD values of hits (Snodgrass and Asiaghi, 1977). Accuracy in percentage was calculated using Equation 1


$$\text{Accuracy}\;(\%)=\frac{H+\mathrm{CR}}{H(\max)+\mathrm{CR}(\max)}\times 100$$ (1)

Where,

H = Number of Hits.

CR = Number of Correct Rejections.

H (Max) = Maximum number of Hits allowed.

CR (Max) = Maximum number of Correct Rejections.

**Figure 2:**
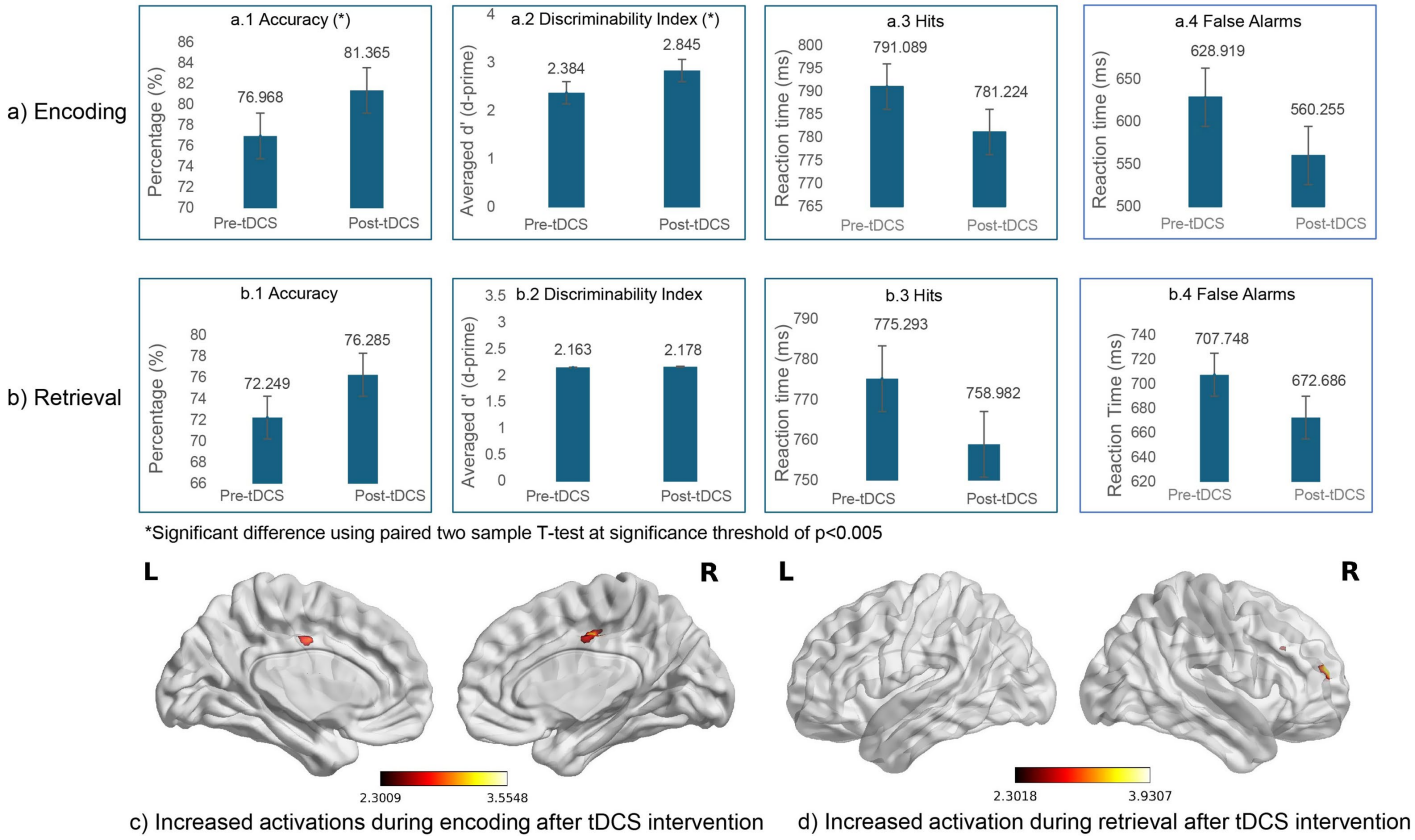
Comparison of performance measures (accuracy, discriminability index, hits, false alarms) during **(a)** encoding and **(b)** retrieval tasks of the episodic memory fMRI paradigm at baseline and following tDCS intervention (pre- vs. post-tDCS) in the patients with early AD (*N* = 30; MCI *n* = 15, mild AD *n* = 15) who achieved an accuracy level of 60% or more at both time points. Significantly increased activations were noted following tDCS during **(c)** incidental encoding (non-living) trial at the bilateral posterior cingulate gyrus and **(d)** the intentional retrieval (non-memory) trial in the right middle frontal gyrus and right frontal pole using clusters determined by *Z>*2.3 and a corrected cluster significance threshold of *p* < 0.05 (Worsley, 2001). All the activation maps are co-registered to MNI template. Cluster coordinates are depicted according to FSL standards and displayed using BrainNet Viewer toolbox (Xia et al., 2013). Refer Supplementary Figures 4, 5 for original outputs from FSL.

Three patients with MCI and five patients with mild AD performed below the predefined accuracy threshold of 60% during the pre- and/or post-tbfMRI acquisitions; these eight participants were therefore excluded from further analyses. Within the MCI group, one participant failed to reach the accuracy threshold during the pre-tbfMRI session, one during the post-tbfMRI session, and one during both pre- and post-tbfMRI sessions. Similarly, within the mild AD group, behavioral data could not be recorded for one participant (the first participant enrolled in the study) during the pre-tbfMRI session, one participant did not meet the accuracy threshold during the post-tbfMRI session, and three participants did not meet the threshold during both pre- and post-tbfMRI sessions. The performance metrics for the remaining participants (*N* = 30; MCI *n* = 15 and mild AD *n* = 15) for the incidental encoding and intentional retrieval tasks are shown in Figure 2. Task-based fMRI data pre-processing and analyses were carried out using FEAT (FMRI Expert Analysis Tool) Version 6.00 of FSL, implementing high-quality model-based fMRI data analysis (Jenkinson et al., 2012; Smith et al., 2004). Z (Gaussianised T/F) statistical images were thresholded non-parametrically using clusters determined by Z > 2.3 and a corrected cluster significance threshold of *p* = 0.05 (Worsley, 2001) (Figure 2; displayed using BrainNet Viewer toolbox (Xia et al., 2013); Supplementary material for original outputs from FSL and Supplementary Flowchart 2).

#### 2.6.4 Resting-state MEG

Two participants (1 in the MCI sample and 1 in the mild AD sample) opted out of taking part in MEG acquisition, and four participants (3 in the MCI sample and 1 in the mild AD sample) did not participate in post-MEG scans; MEG data of the remaining participants (*n* = 34) were preprocessed (Supplementary Table 3 for sociodemographic details of these participants). The analysis of the available pre- and post-rsMEG data from patients with early AD (*n* = 34; 15 in the MCI sample and 19 in the mild AD sample) was performed. Bad channels (<2% of total channels in any given case) were identified during MEG acquisition and were not considered for further analysis. MaxFilter processing, including tSSS was applied as a part of initial preprocessing pipeline. This rsMEG data was imported using Brainstorm® software for data visualization and manual inspection. Subsequently temporal filtering was performed using a high pass filter (linear phase Finite Impulse Response-Kaiser) of 0.3 Hz to remove DC offset, followed by applying a notch filter up to the third harmonics at 50 Hz with 3-dB bandwidth. Additional bad segments were excluded after visual inspection (Francois Tadel et al., 2011). Following tSSS and filtering the signal space projection (SSP) technique was used to identify the sensor topographies due to specific artifacts (e.g., blink and cardiac artifact), and its spatial projectors were generated using Principal Component Analysis (PCA) to remove these artifacts (Uusitalo and Ilmoniemi, 1997). Additionally, Independent Component Analysis (ICA) was used to filter out the frequency components for eye movement, subject movement, artifacts due to dental implants (between 1 and 7 Hz), muscle noise, and sensor artifacts (between 40 and 240 Hz).

For pre-processing the rsMEG data, T1-weighted NIfTI image files from the pre- or post-tDCS MRI sessions with the best QC metrics (see above) were selected. Using FreeSurfer 6.0 software, subject-specific cortical surfaces were reconstructed from these structural MRIs. The pre-processed structural images and the max-filtered MEG data were imported into the Brainstorm® software database, and the subject-specific structural images were used to identify the subject coordinate system. Anterior and posterior commissures and intra-hemispheric points were marked, and each MRI was normalized to MNI coordinates based on the affine co-registration with the MNI ICBM 152 template. Co-registration of the MEG sensors ensured the estimation of source activity with the subject-specific anatomy. Using the overlapping sphere method, forward head modeling was done, followed by source reconstruction using inverse modeling through a linearly constrained minimum variance (LCMV) beamformer advanced source reconstruction algorithm (Tadel et al., 2011). After pre-processing, noise covariance was calculated from empty room recordings to measure the sensor noise, and data covariance was calculated from the resting-state MEG recordings.

Using the Welch method, a power spectrum analysis was performed on 5 min of artifact-free MEG recording. This analysis estimated the power at different frequencies using a window length of 4 s, with a 50% overlap between two consecutive time windows. Extracted power spectrum density (PSD) values for neural oscillations covered canonical frequency bands [delta (2–4 Hz), theta (4–8 Hz), alpha (8–12 Hz), beta (13–30 Hz), gamma 1 (30–60 Hz) and gamma 2 (60–90 Hz)]. Spectrum normalization involved dividing the PSD values by the total power of the participant’s source signal that was spatially smoothed with an FWHM of 3 mm. A paired T-test was performed between the pre- and post-tDCS values of normalized PSD, correcting for multiple [permutation testing (1,000 randomizations), Monte Carlo method] comparisons (FDR-corrected), controlling for time and frequency.

The dependence between the phase of low-frequency oscillations (fP) and the amplitude of high-frequency rhythms (fA) was quantified using PAC of resting-state MEG data. PAC was computed using the Modulation Index (MI) method as described by Tort et al. (2010) and implemented in the Brainstorm® software (Tort et al., 2010; Tadel et al., 2011). To estimate the instantaneous phase of the low-frequency filtered signal and the amplitude of the gamma-band signal, the desired spectral range for fP and fA was selected between 4–8 Hz (theta) and 30–90 Hz (gamma), respectively. The coupling strength for each participant was estimated using time-resolved PAC (tPAC), which shows the least relative error. The tPAC was estimated by searching for the fP oscillation with the strongest PAC to fA bursts, over a time window, which slides on the input MEG data. A 10-s epoch length was used for analysis, and since the fP band of interest was between 4 and 8 Hz, three full cycles of the fP band were chosen to produce a sliding window length of 0.75 s. The average comodulograms were extracted using tPAC maps of all the patients with MCI (*n* = 15) and mild AD (*n* = 19) in the left entorhinal cortex (Jacobs et al., 2007; Rodriguez et al., 2020) which plays a critical role in episodic memory and is vulnerable to early pathophysiology in AD. All the MEG results were generated using Brainstorm® software (Tadel et al., 2011). A paired *t*-test was performed outside this software using MATLAB® (see text footnote 2) to find out the significant (FDR-corrected *p* < 0.05) difference in coupling strength between phase of low frequencies (4–8 Hz) and amplitude of high frequencies (30–90 Hz) correcting for multiple comparisons (*n* = 1911) across time (*n* = 49) and frequency (*n* = 39) (Supplementary material for the original outputs and the analysis scripts).

## 3 Results

The overall sample of participants with early AD (*n* = 40) (Table 1) showed significant improvement in verbal memory [Learning Trial 3 and Delayed Recall) scores following tDCS (post- vs. pre- tDCS (Supplementary Table 1)]. On post-hoc sub-group analysis (MCI, *n* = 19; mild AD, *n* = 21), delayed recall scores showed significant improvements following tDCS, specifically in the MCI sub-group. As evident from Supplementary Table 1, a substantial number of the items in NNB-E showed trends (*p* < 0.05) toward improvement following tDCS, but these differences turned out to be non-significant following correction for multiple comparisons.

**Table 1:** Socio-demographic and clinical details of the study participants.

Socio-demographic and clinical variables	Early AD (MCI & mild AD) (*n* = 40)		
	Overall sample (*N* = 40)	MCI (*n* = 19)	Mild AD (*n* = 21)
Age (years)	68.37 ± 7.23	66.53 ± 6.10	70.05 ± 7.89
Education (years)	13.78 ± 3.52	14.68 ± 3.46	12.95 ± 3.44
Gender (Male: Female)	25: 15	10: 9	15: 6
Number of Languages fluent in	2.90 ± 1.19	3.00 ± 1.20	2.81 ± 1.21
HMSE	26.13 ± 3.16	28.21 ± 2.04	24.24 ± 2.79
EASI	3.90 ± 2.59	2.10 ± 2.02	5.52 ± 1.89

MCI, Mild Cognitive Impairment; AD, Alzheimer’s disease; HMSE, Hindi Mental Status Examination; EASI, Everyday Ability Scale for India.

A significant reduction in seed (*l*MFG)-to-voxel (S2V) rsFC was noted in bilateral posterior cingulate gyri and precuneus following tDCS intervention (Figure 1). An exploratory post-hoc regression analysis of the S(*l*MFG)2 V-rsFC with neuropsychological performance scores was carried out by correlating the NNB-E subtest scores with the subject-wise rsFC values of both the clusters that showed significant reduction in rsFC after tDCS. This analysis revealed a positive relationship of S2V-rsFC with omissions [cluster size: 223 voxels; *r* = 0.356 (95% CI, 0.041–0.607), *t* = 2.2921 (36), *p* < 0.03] and commissions [cluster size: 71 voxels; *r* = 0.349 (95% CI, 0.033–0.601), *t* = 2.2366 (36), *p* < 0.03] on the picture cancellation test and a negative relationship of S2V-rsFC with word list learning trial 3 performance [cluster size: 71 voxels; *r* = −0.374 (95% CI, −0.619-0.061), *t* = −2.419 (36), *p*-value<0.02].

The analysis of behavioral performance during tbfMRI in the sample of patients who performed at an accuracy level of 60% or greater during both the baseline and post-tDCS fMRI acquisitions (*n* = 30), revealed significant (*p* < 0.05) improvement in the accuracy of responses during the incidental encoding task and an overall improvement in the reaction time for both incidental encoding and intentional retrieval trials following tDCS intervention. The discriminability index in the incidental encoding task was noted to improve significantly (*p* < 0.05) following tDCS; however, no significant improvement of the discriminability index was noted in the intentional retrieval task (Figure 2).

Comparison of BOLD hemodynamic responses between post-tDCS and baseline tbfMRI scans (post-pre) in the above sample of 30 patients (MCI *n* = 15; mild AD *n* = 15) revealed significantly increased activations in the bilateral posterior cingulate gyri during “non-living” trials of the incidental encoding task and increased activations in the right middle frontal gyrus (rMFG) and the right frontal pole (rFP) during “non-memory” trials of the intentional retrieval task using a corrected cluster significance threshold of *p* < 0.05 (Figure 2).

**Figure 3:**
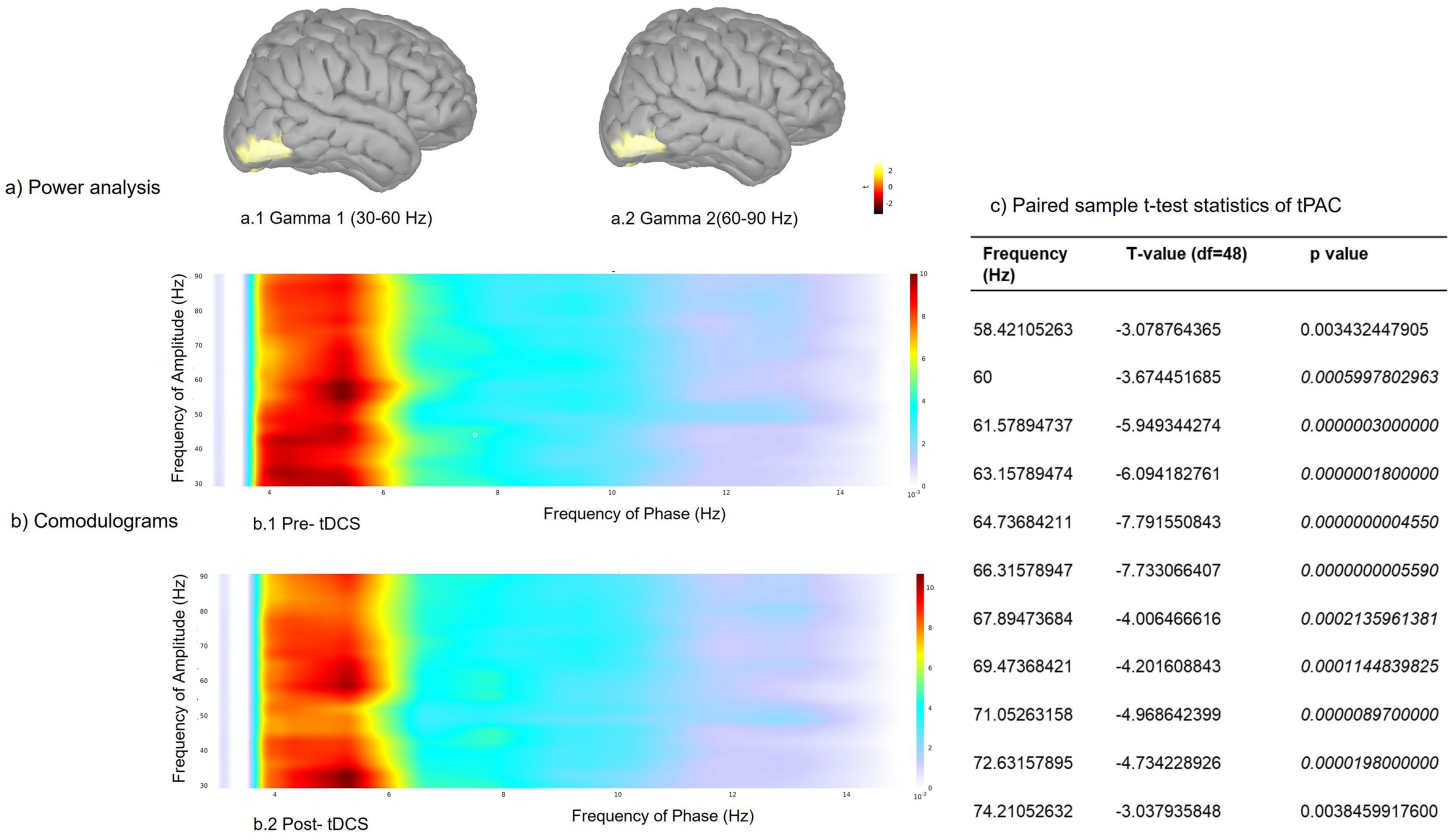
Resting-state MEG analyses comparing normalized power spectral density (PSD; whole brain) and theta-gamma phase amplitude coupling (PAC; left entorhinal cortex) pre- and post- tDCS in patients with early AD (*n* = 34), correcting for multiple comparisons and controlling for time and frequency. **(a)** Significant PSD reductions (FDR corrected *p* < 0.05) were noted following tDCS intervention in the higher frequencies (a.1) gamma 1 (30–60 Hz) and (a.2) gamma 2*n*. **(b)** Comparison of average time-resolved PAC (tPAC) comodulograms between pre- and post- tDCS MEG timeseries (b.1 and b.2, respectively) revealed a significant increase (FDR corrected *p* < 0.05) following tDCS in PAC between the phase of low frequency oscillations (5.30 Hz) and amplitude of high frequency rhythms (60–72.63 Hz), corrected for multiple comparisons. **(c)** T and *p*-values corresponding to those frequencies (6072.63 Hz) showing a significant increase in tPAC post-tDCS (*p* = 0.05/39, i.e., *p* < 0.00128).

Power spectrum analysis of pre- and post-tDCS rsMEG data of patients with early AD (*n* = 34; MCI = 15, mild AD = 19) showed significantly (FDR corrected *p* < 0.05 correcting for multiple comparisons) decreased gamma power in the right occipital cortex after tDCS intervention (Figure 3). The time-resolved phase amplitude coupling (PAC) between rsMEG signals showed that the coupling between the phase of low frequency (theta) and amplitude of high frequency (gamma) increased significantly (FDR corrected *p* < 0.05 correcting for multiple comparisons) after tDCS intervention in the left entorhinal cortex (Figure 3).

## 4 Discussion

The present study aimed to investigate brain functional alterations accompanying the cognitive modulatory effect of anodal tDCS in patients with early AD, using resting-state fMRI and MEG as well as task-based fMRI. We observed significant improvement in verbal memory following anodal tDCS of the left DLPFC in patients with early AD. This was accompanied by a reduction in seed(lMFG)-to-voxel rsFC in the bilateral precuneus and PCC, increased tbfMRI BOLD activations in bilateral PCC and rMFG/rFP during encoding and retrieval tasks, respectively, decreased rsMEG gamma power in the right occipital cortex as well as increased resting theta-gamma PAC in the left entorhinal cortex. These findings provide mechanistic insights regarding the cognition-enhancing effect of anodal tDCS in early AD.

Significant improvement in verbal learning and delayed recall was noted in our early AD sample following anodal tDCS at the left DLPFC. Trend-level improvements were noted in other domains as well, including logical memory, sustained attention, planning, and calculation (Supplementary Table 1; Supplementary Figure 3). In addition, significant improvements were noted in the response accuracy and discriminability index during incidental encoding task performance of the tbfMRI paradigm following tDCS. Trend level improvements were noted post-tDCS in the accuracy and discriminability index during the performance of the intentional retrieval task. The cognitive enhancement noted in the present study, especially in verbal learning, delayed recall, and other domains following anodal tDCS at left DLPFC, replicates the findings of previous studies carried out in patients with MCI and mild AD (Gomes et al., 2019; Murugaraja et al., 2017; Im et al., 2019; Khedr et al., 2014) and provides strong support for its potential as an effective intervention for cognition enhancement especially during the early phase of AD. Various mechanisms, including increased dopamine release (Fukai et al., 2019), cortical excitability and neuroplasticity (Chen et al., 2022) have been proposed to mediate the above cognition enhancing property of anodal tDCS.

A reduction in S(lMFG)2V-rsFC with bilateral PCC and precuneus was noted in patients with early AD following tDCS intervention. Recent evidence suggests that the precuneus and posterior DMN regions exhibit network hyperexcitability and aberrant synchronization in AD, which aligns with our finding of reduced rsFC between lMFG and PCC/precuneus in patients following tDCS (Casula et al., 2023; Giorgio et al., 2024). We previously reported increased rsFC in the executive network in patients with AD (Joshi et al., 2019) and MCI (Bharath et al., 2017), which was interpreted as a compensatory functional response of the relatively preserved neurons to the cortical neuronal loss and white matter disintegration in early AD (Joshi et al., 2018; Qi et al., 2010). This compensatory hyperconnectivity may be offset by the neuroplastic beneficial effects of tDCS, leading to improved cognitive performance. Such posterior DMN regions, including the precuneus and PCC, have also been highlighted as potential targets for non-invasive brain stimulation interventions in AD (Boggio et al., 2022; Koch et al., 2022). The PCC is a key component of DMN, which regulates attention, internally directed cognition, spatial memory, configural learning, and retrieval (Leech and Sharp, 2014; Maddock et al., 2001). Precuneus, another core unit of the DMN, is involved in highly integrated tasks like visuospatial imagery, episodic memory, and retrieval (Cavanna and Trimble, 2006). A decrease in PCC-precuneus rsFC was linked, in a recent study by Wu et al. (2023), to the improvement in immediate-recall ability in patients with MCI who were trained to enhance their episodic memory. Thus, the reduction of PCC-precuneus rsFC observed in the present study can be linked to the pro-cognitive effect of tDCS, which in turn attenuates the compensatory functional hyperconnectivity in early AD.

We performed a post-hoc regression analysis of the S2V-rsFC with neuropsychological performance scores to further examine the link between rsFC and cognition, which revealed significant positive correlations between the rsFC values at PCC and precuneus with the omission and commission scores of the picture cancellation task, respectively; and a negative correlation between rsFC values of both the PCC and precuneus with performance on learning trial 3. These findings along with the recent observations by Wu et al. (2023) provide compelling evidence for decreased rsFC involving bilateral PCC and precuneus as imaging markers of the cognition-enhancing effect of anodal tDCS in early AD.

The tbfMRI results showed increased activations following tDCS intervention in bilateral PCC during the performance of the incidental encoding task; and in the rMFG and rFP during the intentional retrieval task. This may reflect the normalization of brain functioning following anodal tDCS by enhancing the prefrontal cerebral blood flow (Das et al., 2019; Chan and Han, 2020). However, these increased task-related activations may reflect enhanced neural efficiency, compensatory recruitment of additional neural resources, or a combination of both, particularly in the context of neurodegenerative disease. Given the limited sample size for tbfMRI analysis, increased activation in bilateral PCC was seen only with the “non-living” trials of the incidental encoding task, whereas increased activations in the rMFG and rFP were noted only with the “non-memory” trials of the intentional retrieval task. The findings from this initial study that has used tbfMRI to explore the effect of anodal tDCS on brain activations during episodic memory encoding and retrieval may, therefore, be considered preliminary, and indicate improved activation of brain regions involved in spatial information processing (Rolls, 2019), episodic memory and recognition (Huijbers et al., 2011; Papma et al., 2017; Wagner et al., 2005) during encoding as well as regions involved in attention, working memory and prospective memory (Japee et al., 2015) during retrieval.

The rsMEG results revealed a significant reduction in gamma power in the right occipital cortex following tDCS intervention, along with a substantial increase in theta-gamma PAC in the left entorhinal cortex. This is the first demonstration of altered rsMEG markers of improved cognitive performance (Victorino et al., 2023) following anodal tDCS intervention in patients with early AD. Though the causal mechanisms by which the gamma rhythms influence specific brain functions remain unclear, it is widely understood that the different bands of gamma oscillations serve an orchestration function to synchronize neuronal populations that are involved in various brain functions in space and time (Fernandez-Ruiz et al., 2023). It follows therefore that an increased level of synchrony does not necessarily imply a computational advantage (Fernandez-Ruiz et al., 2023), and could in fact represent an aberrant pattern of connectivity in pathological states. Increased gamma-band power (van Deursen et al., 2008) and gamma-band coherence (Başar et al., 2017) have been reported in AD and MCI and may thus reflect an effortful compensatory inter-regional integration in the face of the progressively evolving neurodegeneration in early AD. These alterations in gamma-band activity are interpreted as disrupted or impaired gamma oscillatory dynamics. Anodal tDCS has been shown to have strong remote gamma band effects in cortical regions that are located far away from the site of stimulation (Pellegrino et al., 2019). Thus, the reduction of right occipital gamma power in the present study reflects the mitigating effect of anodal tDCS at left DLPFC on aberrant gamma-band activity in patients with early AD.

The PAC of the rsMEG, an index of how high-frequency oscillatory activity is organized according to the phase of a low-frequency oscillation, is critical for multiple components of episodic memory. The results of our study demonstrate for the first time how anodal tDCS intervention significantly increases theta-gamma tPAC in the left entorhinal cortex in early AD, commensurate with significant improvement in episodic memory. Increased cross-regional PAC between the entorhinal cortex and parahippocampal cortices has been linked to successful episodic memory encoding using intracranial EEG (Onslow et al., 2011; Wang et al., 2021). This finding underlines the significance of cross-frequency coupling (CFC) in cognitive performance (Fernandez-Ruiz et al., 2023) and points toward how the enhancement of CFC in the left entorhinal cortex following anodal tDCS, a downstream effect, leads to improved verbal learning and episodic memory in early AD.

To the best of our knowledge, this is the most comprehensive study to date that has attempted to examine the mechanistic basis of cognitive enhancement following tDCS using multiple modalities of functional brain imaging. The findings of the study highlight a very interesting synergy between the fMRI and MEG results. The improved cognitive performance following anodal tDCS intervention in early AD was accompanied by an increase in theta-gamma tPAC of rsMEG in the left entorhinal cortex, which indicates improvement in CFC essential for cognitive performance, as well as increased tbfMRI BOLD activations of critical brain regions involved in the performance of encoding and retrieval. Furthermore, rsfMRI and rsMEG markers that reflect the compensatory functional responses of the relatively preserved neurons in early AD - namely, increased rsFC and increased gamma power, respectively, were reversed following anodal tDCS. Overall, this intervention in combination with other treatment strategies such as pharmacology, cognitive re-training, etc., has the potential to significantly improve cognitive performance and help in the maintenance of these improvements over a longer time duration.

Several limitations of the present study should be acknowledged. First, the absence of a sham or control group limits this study in interpreting whether the observed changes can be attributed specifically due to the effects of tDCS, rather than to nonspecific factors such as differences in arousal or engagement, practice or placebo effects, or the natural course of the illness. Second, though we report our findings on a sizeable sample of patients with early AD in comparison to many previous studies (Inagawa et al., 2019; Ladenbauer et al., 2017; Hanley et al., 2016), the challenges in carrying out such a comprehensive study on elders with cognitive impairment during the COVID pandemic period meant that we only managed to complete the study in a much more limited sample than was originally planned. Third, despite recruiting a comparatively larger cohort than many prior studies, modality-specific attrition occurred across resting-state, task-based fMRI and MEG analysis due to data quality issues (e.g., motion artifacts), incomplete acquisitions, task performance below predefined accuracy thresholds, and participant tolerance or availability for MEG sessions. This attrition resulted in differing sample sizes across imaging modalities, which may have reduced statistical power and limited the generalizability of multimodal convergence. Fourth, the limited sample size also precluded separate analyses of the functional brain changes in the MCI and mild AD samples. Fifth, we did not attempt to control for the influence of confounding factors such as comorbidities and medications. Finally, we recruited our samples based on stringent diagnostic criteria as detailed earlier; however, we did not take into account the more recently suggested biological framework of the NIA-AA for the diagnosis of AD (Jack et al., 2018). Nonetheless, this is unlikely to have affected our findings, as recently reported by Kang et al. (2021) in their study on the impact of tDCS on cognition in patients with MCI.

## 5 Conclusion

The findings of this resting fMRI/MEG and tbfMRI study provide mechanistic insights regarding brain functional alterations that underlie the cognitive modulatory effects of anodal tDCS in early AD. The enhancement in episodic memory performance following anodal tDCS of the left DLPFC was noted to be accompanied by alterations of rsMEG and tbfMRI markers that reflect enhanced cross-frequency coupling and regional task-related brain activations respectively, as well as reversal of rsfMRI and rsMEG markers that indicate compensatory functional responses of the relatively preserved neurons in early AD. These have the potential to be considered as markers of treatment response to NIBS procedures in MCI and AD.

## Data Availability

The raw data supporting the conclusions of this article will be made available by the authors, without undue reservation.

## Footnotes

1
https://github.com/rordenlab/dcm2niix

2
https://www.mathworks.com

## Author’s note

This work was previously presented in part as a poster at the Alzheimer Association International Conference (AAIC), held in Amsterdam, The Netherlands, July 16–20, 2023. The abstract was published in an online supplement to Alzheimer’s & Dementia: The Journal of the Alzheimer’s Association. Joshi H. et al. The role of transcranial direct current stimulation in improving learning and memory performances of patients with mild cognitive impairment and mild Alzheimer’s disease: a functional magnetic resonance imaging study (Joshi et al., 2023).
